# Academic Success at Social Costs: An Exploratory Study on Social Networks of Chinese Students under Academic Streaming

**DOI:** 10.3390/ejihpe14010011

**Published:** 2024-01-04

**Authors:** Jinjing Fang, Gavin T. L. Brown

**Affiliations:** Faculty of Education and Social Work, The University of Auckland, Auckland 1142, New Zealand; gt.brown@auckland.ac.nz

**Keywords:** social networks, mixed methods, academic streaming, assessment, higher education

## Abstract

In universities that require students to reside in dormitories, there are two types of social networks—study/classroom-based and social/dorm room-based. The academic streaming system may disrupt study/classroom connections, but its impact on students’ social networks is unknown. Using self-reported surveys, this study examines ego network measures of network sizes, turnover, multiplexity, and diversity among 382 students (44% female, 56% male). Surveys were administered before and after the university employed a first-semester grade-point average to demote or promote students into an honours college. Follow-up interviews were conducted with 11 honours students staying within their track and 11 students who were re-streamed to the non-honours track. Quantitative results showed that students in the non-honours college and who remained there had increasingly overlapping friendship circles between study and social environments, along with more diverse social connections, indicating stronger networks. In contrast, honours participants experienced fewer overlapping networks across domains and less dispersed social ties, especially after the academic replacement process. Qualitative results showed that the honours students faced a trade-off between academic success and social engagement in maintaining their elite status. Re-streamed students experienced otherness in social groups and decreased psychological wellbeing. This study contributes to the application of network analysis in education and provides insights into the unintended consequences of educational practice on students’ social networks.

## 1. Introduction

Social networks exert considerable influence on students’ academic behaviours, performance, and psychological wellbeing [1,2,3]. In institutions that require students to reside in dormitories, students’ networks with peers in both study and living environments play a significant role in shaping their experiences [4]. Stable and diverse networks across settings provide resilient social support, whereas unstable and isolated networks can be harmful to wellbeing [5,6].

Educational policies (e.g., streaming, selective admission) may reweave students’ social networks by changing classes and circumstances where they build connections [4]. Despite the prevalence of streaming in secondary [7] and higher education [8], little is known about its impact on students’ social networks in academic and non-academic contexts. Hence, this study aims to investigate students’ relationship patterns and how they are influenced by institutional streaming. Through ego network analysis and interviews, this research sheds light on the unintended consequences of selection/relegation mechanisms on the network dynamics and experiences of high-achieving learners.

### 1.1. Peer Networks in Higher Education

In educational research, peer networks take on the characteristics of social capital that serves various purposes, including the successful attainment of achievement goals [9,10]. Positive peer networks in classrooms can help foster students’ positive self-beliefs, prosocial–cooperative behaviours, wellbeing, and motivation [11]. Additionally, when interacting with their new college peers, particularly roommates, students establish essential social resources that shape their academic experiences and outcomes [4,12,13,14,15].

The importance of roommates becomes greater when institutions assign students to dormitories and compel them to be in dormitory housing for at least the first year of university. This stands in contrast to the experience students have when they are allowed to choose where and with whom to live, which might be seen in commuter campuses or when post-freshmen are allowed to live out of dormitories. Despite the debate over whether college roommates significantly influence achievement, consistent evidence has been found for peer effects of roommates on individual behaviours such as social activity participation, academic choice, academic major, and job selections [14,15,16,17]. Therefore, determining peer relationships not only at the classroom level but also at roommate or dorm levels should give a reasonable approximation of personal social networks in higher education [17].

### 1.2. Effects of Educational Policies on Peer Networks

By changing how students are streamed into academic classes from their initial arrangement, institutional decisions can re-configure students’ social networks, generating different peer effects [4]. Sorting strategies (e.g., academic tracking or selective admissions) are used by policymakers and administrators with the intention of amplifying positive effects on students’ academic achievement, especially for those with a higher ability [4,12,18]. However, this practise has more negative than positive consequences, especially for students in lower-ability streams [7]. Yet, research has yet to deeply understand how academic streaming may shape students’ social networks.

Among limited studies, Hallinan and Sørensen [19] conducted a longitudinal investigation examining the impact of streaming group membership on friendship formation. Students in the same ability group were more likely to choose friends of similar characteristics due to their interactions and shared experiences. The increasing density of friendship over time underscored more existing similarities (e.g., similar reading ability) and created greater similarities among norms, values, and attitudes. Their results correspond to the prominent findings of homophily in social network analysis in that individuals tend to form connections with similar peers [20,21].

Recent research also suggests that academic streaming has implications for students’ social capital in their networks. Peer relationships within a high-achieving group can offer students the opportunity to establish shared norms, values, and aspirations, which may result in improved educational outcomes, including enhanced academic growth and increased success in the job market [22,23,24,25]. Nevertheless, the practice of sorting students according to their academic aptitude does not consistently yield advantageous results. It is possible that such grouping could lead to an inclination towards forming exclusive social groups or cliques among students (i.e., social closure) [26], as there are limited opportunities for students to interact with peers outside of their academic class. Individuals in segregated environments may experience limited access to supportive networks, social engagement opportunities, and participation in a wider community [23,27].

In light of these mixed findings, it is imperative to investigate the potential impact of academic streaming on students’ social networks. It can be argued that the practice of grouping students in an honours class would lead to (a) the development of an elite identity, rooted in a perceived superior academic status [26,27,28,29], (b) maladaptive social comparisons against members of their own stream and those of other streams that prevent students from developing a realistic assessment of their own abilities [7,30,31,32], and (c) the development of unreliable relationships in a competitive environment [18,27,33].

### 1.3. Peer Relationships of Chinese Students

Similar to the results obtained from Western countries, peers are a significant context for Chinese student learning and personal development [18,34]. Adaptive peer relationships have positive effects on Chinese learners’ academic performance, motivation, and social functioning (e.g., social competence and adjustment) [1,10,35]. In Confucian heritage societies like China, the importance of social relations and interactions with significant others (e.g., parents, teachers, and peers) is woven into the fabric of their educational experience [3]. Pressure from students’ social groups or society in general may motivate students to exercise strong effort to obtain social approval or fulfil familial obligations [36,37,38]. In particular, peers can serve as a comparative source of achievement motivation that drives Chinese students to learn [39]. Adopting performance-related goals (i.e., outperforming others in academic settings) may help students avoid the disappointment, guilt, and shame that arises in the eyes of significant others from underachievement [39,40]. However, emphasis on examination, achievement, and competition may also generate competitive attitudes and internal and peer pressure that are maladaptive for students’ psychological wellbeing [18,35].

Further, in Chinese higher education, residential-based peer relationships play a significant role because dorms and rooms are the centres of students’ social interactions [16,41]. Frequent contact, shared activities, and interpersonal affective connectedness can provide supportive and comparative sources of social processes that affect students’ behaviours, learning processes, and outcomes [1,18]. Maladaptive competition in academic settings, on the other hand, can work against positive peer influence and network construction [18,33]. Especially for students in elite universities, there may be general reluctance to share insights about learning challenges or even let peers know about their learning difficulties. This applies even to roommates living in close proximity because such an admission could hurt their chances of beating their competition and obtaining rewards (e.g., scholarships, opportunities) [18]. It should be noted, however, that the majority of research on Chinese students’ peer relationships has focused on elementary and secondary education, particularly in classroom settings [10,35]. Regarding the significance of the residential context in higher education, it is necessary to consider both class- and dorm room-based environments when examining student social networks.

Understanding Chinese students’ social networks becomes particularly important when academic streaming comes into play. When an educational policy alters students’ placement in streams based solely on their academic performance without regard for their social environment, social networks may be affected [4,23,31]. Recent research has analysed the effects of academic streaming on students’ academic performance [31], psychological wellbeing [29,31,32,42], and social integration [29]. However, few studies have focused specifically on the social networks of Chinese students under streaming, although this practice is increasing in popularity in China [8]. Furthermore, studying the social networks of Chinese students under academic streaming is meaningful because the Chinese education system is characterised by its highly competitive nature and emphasis on academic achievement [33,39]. Understanding how streaming influences students’ social connections in this context can provide insights into the broader social and psychological implications of educational tracking practices [24]. Thus, this study uses social network analysis based on ego network measures to examine the impact of academic re-streaming on students’ social connections.

### 1.4. Theoretical Foundation: Ego Network Analysis and Measures

An ego network is a kind of social network that composes a central actor and all his or her relationships with other persons (called alters), as well as the relationships among these alters [43]. Rather than focusing on a particular group or a whole network, ego networks are networks that focus on one actor whose networks can be characterised by *connections* between alters in contrast to *positions* between them nested in a whole network [44]. Thus, ego network analysis emphasises the individual in the context of their relationships as the unit-of-analysis.

Ego networks can be described by the number (i.e., network size) and nature (i.e., multiplexity) of relationships between egos and alters (i.e., other members of a social network), alter attributes (i.e., their heterogeneity or homogeneity), and network structure (i.e., patterns of relationships among alters) [45]. Specifically, the larger one’s social network, the greater adaptive social support one can anticipate receiving [45,46,47]. A more wide-reaching social network is associated with better wellbeing [48], but maintaining it may have a negative impact on wellbeing due to information overload or negative social exchanges such as failure to provide help and insensitive behaviour or rejection, even in the case of positive ties [49].

Multiplexity in social networks has a beneficial impact on individuals as well [43,44,50]. The degree of multiplexity indicates the extent to which members of a network are involved in a variety of settings (e.g., studying in the same class, living in the same dorm room, or shared socialising outside class or the dorm) [51]. More motivation for relationship maintenance is present in multiplex networks because egos interact with alters in a variety of contexts that may elicit greater intimacy, support, and trust [20,43,50,52].

Additionally, greater ego–alter similarity may lead to stronger network formation [45,53] because individuals prefer to establish positive connections with those who are like themselves (i.e., homophily) or with whom they have easy access (e.g., study group or dorm room). Moreover, repeated interactions help people develop similar behavioural or attitudinal norms, which contributes to the formation of closer relationships and greater homophily [54].

Networks with more heterogeneity (e.g., network variation) may reap more benefits because a greater variety of cross-boundaries networks suggests more diverse patterns of interaction or integration of ideas and resources [45,46]. The increasing worth of students’ social assets and resources could help them better target and access information via networks, leading to improved academic outcomes [2,13].

Considering longitudinal data, network dynamics can be measured by network turnover [45]. Turnover is a way to understand how ties would be dropped from or added to networks when personal or environmental circumstances change over time [5,55]. If access to strong ties is lost or stable ties are weakened, particularly without the replacement of alters (i.e., high membership turnover), individuals may experience decreased wellbeing [45].

This theoretical framework is suited for understanding the social networks of students under academic streaming. First, it allows for a thorough examination of the structure and dynamics of individuals’ social connections within a specific context [43]. By focusing on the ego and its immediate network connections, we can gain a deeper understanding of how academic streaming may affect the formation and maintenance of social relationships over time and selection/reselection processes [45]. Secondly, ego network analysis provides insightful information regarding the characteristics of social networks. It enables the use of a variety of measures, such as network diversity and homophily, that help us identify influential factors affecting students’ network dynamics [23]. Additionally, in previous research, ego network analysis has been increasingly applied to educational settings [23,45]. Scholars have utilised this methodology to investigate various facets of students’ social relationships, including peer influence [18,33,36,38] and social support [22,46,47]. By using this established framework, we can contribute to an understanding of how academic streaming may impact the social relationships of Chinese students.

### 1.5. The Present Study

The context for this research is one of the most selective universities in China [8,56,57], enrolling about 6000 students each year. Entry is permitted via performance on the National University Entrance Examination (i.e., *gaokao*), with a minimum score of 670 out of 750 being required. The top 10% of new students are enrolled in an honours programme in an honours college upon enrolment. It is worth noting that this situation of an honours college within a university is somewhat common in China, with approximately 6% of PRC higher education institutions having such a structure (*n* = 77) [8].

At the end of the first semester, high-performing non-honours students are promoted to the honours college (about the top 1% of the non-honours cohort), while honours students who fail to meet the minimum academic requirement (i.e., grade-point average [GPA] ≥ 3.2/4.0, approximately the bottom 10% of the honours cohort) are transferred to the non-honours stream. Note that this re-sorting takes place every semester, but this study focuses just on the impact of the first semester.

In contrast to the possibility of academic movement each semester, dormitory room assignment does not allow for much movement. At enrolment, students in any one faculty are randomly assigned to rooms in dormitory buildings, with honours students assigned to rooms in a dedicated dormitory building. Each room contains four to six students depending on room sizes. Further, the university does not easily permit students to switch rooms after initial assignment [18]. Even after the selection/relegation process at the end of the first semester, students cannot change their rooms until starting their junior (3rd) year when the cohort moves to another campus. 

Therefore, the university setting and procedure involve academic restreaming while keeping dorm assignment stable. Honours and non-honours students who remain in their own stream are likely to maintain their existing social connections within their curriculum group. For students who are moved to a different stream, adjustments to their social networks are likely. Honours students moved down to a non-honours stream might experience a sense of loss in terms of academic recognition or face, as well as a change in classmates with whom they can form social networks. In contrast, non-honours students who are promoted to the honours stream could experience a positive shift in their academic identity and the opportunity for enhanced learning experiences, but they also face a change in classmates with whom they can form social networks. In both cases, movement to a different academic environment may require adaptation to new teaching styles, expectations, and peer dynamics. Discrepancies in educational experiences could potentially impact their social networks, self-perceptions, motivations, and emotions.

The literature has identified three areas of research that serve as the basis for this study. Theoretically, there is a lack of empirical evidence from ego network analysis that investigates the processes and dynamics of students under academic streaming. In terms of methodology, research that has utilised mixed-method approaches from a network perspective is limited. This approach is crucial to obtaining a more comprehensive understanding of the social networks of Chinese students in academically streamed environments. Regarding practical implications, there lacks research investigating the potential social and psychological consequences of academic tracking on students’ experience. Understanding the unintended impact of the streaming mechanism is essential to the decision making of policymakers, educators, and other stakeholders.

Based on ego network measures and analysis, four hypotheses are proposed:

1. Sizes of strong ties are expected to remain similar over time as students invest comparable amounts of energy and effort into relationship maintenance [6,49].
2. Due to the non-alignment of class and dormitory assignment, students experiencing streaming may have distinct networks in academic and social settings, resulting in different network multiplexity.
3. Given the exclusive nature of their class, honour students are likely to have different alter attributes (e.g., more similarity) and network variations.
4. Due to the academic sorting system, students experiencing streaming would demonstrate different network turnover patterns.

## 2. Materials and Methods

### 2.1. Procedure and Participants

Participants were chosen from a cohort of first-year students in the College English course during Semester 1. The survey was administered during the fifth week of both the first and second academic semesters. To enhance convenience and accessibility, online surveys were used as the primary method of data collection. Before initiating the survey, participants were provided with an information sheet that outlined the objectives and methodologies of the study, along with consent forms for their voluntary participation. Each survey took approximately 10 min. Upon the completion of the Semester 2 survey, respondents were presented with the opportunity to volunteer for a follow-up interview.

A total of 742 freshman students volunteered for repeated measures questionnaires approved by the authors’ institutional review board (reference 021664). Table 1 shows the demographics of the sample with matched data (*n* = 382, response rate = 51.5%) after removing missing data (*n* = 5) and duplicates (*n* = 7). We used a chi-square test to evaluate whether there were significant differences in the proportion of participants across groups and conditions. The ratio of non-honours participants to honours students was about 75:25, a statistically significant proportional difference in favour of the latter compared to enrolment (χ^2^ = 7.79, *p* < 0.01). Males outnumbered females in honours classes (χ^2^ = 4.67, *p* = 0.03). The distribution by faculty was very different (χ^2^_(6)_ = 41.60, *p* < 0.001), with more Engineering students in non-honours classes and many more Science faculty students in honours classes. Of the matched sample, six honours students (6%) moved downward, and none were promoted in Semester 2, matching the proportion of students with movements in the population (χ^2^ = 1.09, *p* = 0.30 and χ^2^ = 2.88, *p* = 0.09 for demoted and promoted students, respectively). Honours and non-honours groups have quite different demographic characteristics, so generalisations between them should be read with these differences in mind. The total sample provides sufficient power (1 − β = 0.999) to detect differences between groups on repeated measures with an effect as small as *f* = 0.10.

As the six re-streamed students only represent a small sub-sample, they were excluded in between-group comparisons. However, four of them agreed to participate in follow-up interviews, which were incorporated into data analysis. Additionally, a snowball technique [58] was used to find potential participants being re-streamed to non-honours classes by asking these respondents to introduce the study to their acquaintances who met the criteria. Altogether, 11 out of 30 demoted students (23.33%) and 11 of the students staying in the honours group (12.46%) were interviewed. There was no overlap between alters named by the egos in the interview.

### 2.2. Measures and Analysis

#### 2.2.1. Quantitative and Qualitative Measures

To collect quantitative data of ego networks, we conceptually distinguished four sources of ego friendship (i.e., class, dormitory, room, and other contexts such as student clubs) in two major university life scenarios (i.e., study and social). Network boundaries were specified by defining activities for purposeful actions to clarify tie functions [59]. Strong ties were considered as “best peer friend(s)” who are likely to provide social support (e.g., emotional aid, companionship) [47].

Ego network measures were based on ego–alter ties and alter attributes. Ego–alter ties were analysed by two components (i.e., network size and multiplexity). Network size describes how many strong ties students report with unique alters pooled across situations. Names mentioned as both a study friend and a social friend were not double counted. Multiplexity represents overlaps between tie functions (i.e., study and social friends are the same people), with higher values indicating affectively stronger relationships and higher motivation for relationship maintenance [20].

Agresti’s Index of Qualitative Variation (IQV) was used to measure network diversity [60]. It shows how evenly students’ networks were spread across sources of friendship. It is a standardised measure, where “1” indicates that all alters are evenly dispersed across the four sources of friends and “0” means all cases are in one source. Connections from multiple sources mean a greater range of experiences and support [45].

Network turnover was calculated to examine network dynamics over time [45]. It is a proportional evaluation of network instability or stability as a function of changes in social networks. The number of alter changes (i.e., dropped and added) and retentions are aggregated and divided by the total number of unique alters pooled across both waves.

Regarding the qualitative data, semi-structured interviews were recorded and lasted for approximately 30 min each. Interviews aimed to explore students’ assessment and re-streaming experiences and how these could influence their social networks, motivations, and emotions without prior knowledge of their survey responses. Sample interview questions included:

1. Could you please describe your assessment experience in the university thus far? What motivates you to perform well in assessments?
2. How do you feel about the process of streaming/re-streaming? Does it have any impact on your learning and/or social life? If so, how?
3. Could you share an example of a time when your assessment experience influenced your interactions with peers or friends?
4. Have you noticed any changes in your social network or relationships due to the implementation of assessments or re-streaming? What are they and how do you feel?

#### 2.2.2. Data Analysis

A mixed ANOVA was performed to evaluate differences between groups at each time and within groups across time. Effect sizes were converted to standardised mean differences in Cohen’s (1988) *d* [61].

Thematic analysis [62] was conducted to analyse the transcripts and identify themes without considering pre-existing coding structures. This approach is appropriate when prior literature on a phenomenon provides minimal guidance [63]. Through searching for, reviewing, sorting, and defining themes [62], we developed a thematic map as a visual presentation.

## 3. Results

### 3.1. Quantitative Results

**Figure 1:**
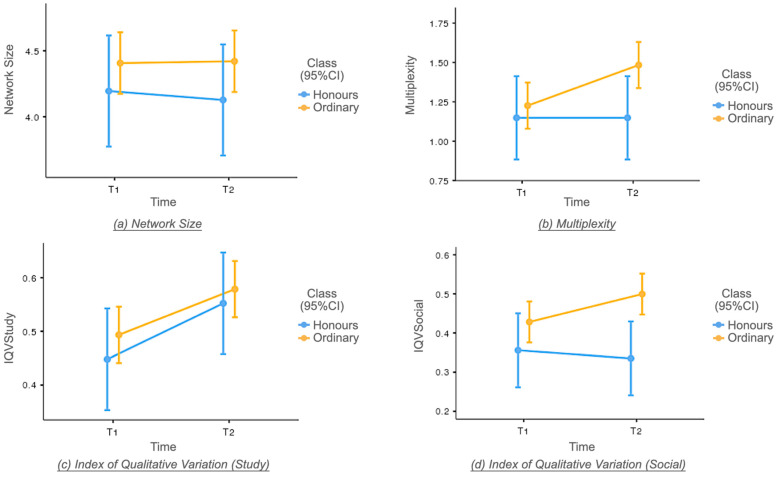
Plot of network measures mean scores across time.

Quantitative results are presented in terms of between-group comparisons and within-group comparisons for honours and non-honours groups, followed by findings from the demoted students. Overall, trivial differences in ego network measures existed between groups in Semester 1 (i.e., Cohen’s *d* < 0.20; see Table 2). However, in Semester 2, the non-honours group increased on all four measures, while the honours group increased only on the study Index of Qualitative Variation (IQV; see Figure 1).

#### 3.1.1. Between-Group Comparisons

In Semester 1, no statistically significant differences between the two groups were observed, meaning that students’ networking patterns did not differ upon admission after gaokao. However, noticeable differences between classes emerged in Semester 2 in terms of multiplexity and social IQV. Specifically, compared to their non-honours counterparts, the honours group had fewer connections that shared study and social settings (multiplexity *F*_(1, 377)_ = 4.75, *p* < 0.05, |*d*| = 0.30). This suggests a distinct separation between environments after re-streaming. Further, honours students’ social networks displayed reduced diversity (social IQV *F*_(1, 377)_ = 7.51, *p* < 0.01, |*d*| = 0.37), indicating that their social networks were more similar in Semester 2. There probably was greater homophily (i.e., bonding with similar ones within class/room/dorm) and homogeneity (i.e., groups of peers that are all honours students) in their connections.

In terms of changes in network turnover between semesters, both groups exhibited comparable patterns. A slightly elevated turnover within the learning environment was evident, indicating a more active replacement of lost ties by new connections in study-related scenarios over time. Both groups had similarity of replacement rates. These findings from between-group comparisons underscore the dynamics of social network restructuring during academic transitions in the first year and highlight the specific alterations that honours students experienced in Semester 2.

#### 3.1.2. Within-Group Comparisons

The within group comparisons showed only one statistically significant but trivially small increase in study IQV for both groups (honours students: *F*_(1, 88)_ = 3.50, *p* < 0.05, |*d*| = 0.20; non-honours students: *F*_(1, 286)_ = 7.60, *p* < 0.01, |*d*| = 0.13). This finding suggests that, irrespective of the class distinction, students’ study networks exhibited a slightly more equitable distribution across various sources of friends. This phenomenon may contribute to a more conducive learning environment characterised by the reduced formation of cliques through transition in classmates. 

In addition, the non-honours students had a statistically significant but trivially small increase in multiplexity values in Semester 2 (*F*_(1, 286)_ = 8.73, *p* < 0.05, |*d*| = 0.20), reflecting somewhat greater tie strengths (Mesch & Talmud, 2006 [50]). This suggests that the non-honours group had more friends who shared social and study experiences. Additionally, the non-honours group had slightly more social relationship variations in Semester 2 (social IQV *F*_(1, 286)_ = 4.66, *p* < 0.05, |*d*| = 0.15). This finding underscores the increasing diversity of their social connections during their first year, suggesting a broader range of interactions and engagement.

#### 3.1.3. The Special Case of Demoted Group

Because of the small sample size (*n* = 6), it is highly likely that the observed effect sizes do not necessarily apply to all demoted students. A close examination of the measures (Table 3) revealed several interesting patterns. Four indicators (i.e., network size, multiplexity, study IQV, and social IQV) had higher values on average across semesters. Four cases (66.67%) consistently had a high variation in study networks (study IQV ≥ 0.84) in both semesters, indicating a tendency to study with friends who were not their classmates or roommates. More dispersed social networks (social IQV ≥ 0.75) in Semester 2 were observed in four cases (66.67%), suggesting that these students were seeking more social support. High disturbance in study networks (turnover > 0.50) for almost all participants of this group (*n* = 5, 83.33%) was expected, as demoted students changed academic tracks from the honours class to the non-honours stream.

### 3.2. Qualitative Results

**Figure 2:**
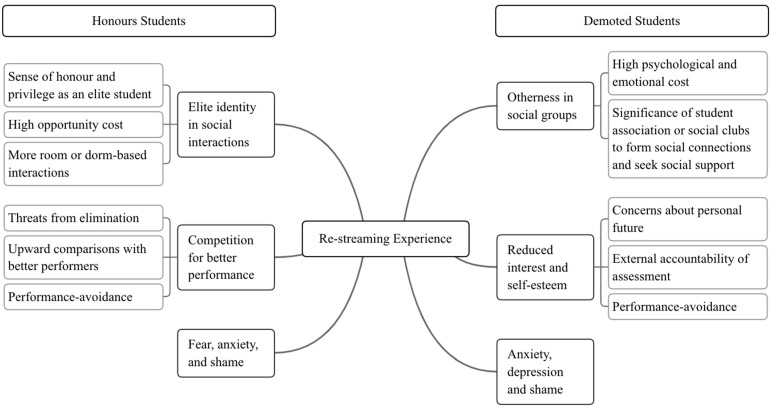
Thematic maps of restreaming experience for honours and demoted students.

Thematic analysis identified three main themes for demoted students (i.e., otherness in social groups, reduced interest and self-esteem, and negative emotions of anxiety, depression, and shame) and three main themes for honours students (i.e., elite identity in social interactions, competition for better performance, and negative emotions of anxiety, fear, and shame). Below are exemplary quotations of students who reacted to their assessment and re-streaming experiences for each of the three themes. Figure 2 shows sub-themes nested within key themes for each group. Path lines show node associations within each group. The bracketed nodes are subthemes related to the superordinate node.

#### 3.2.1. Honours Group

Honours students’ social network patterns revolved around their elite identity (*n* = 11, 100%). One student described ‘the superior status brings a sense of honour and connects us as an elite family’. Another student commented ‘I am surrounded by honours students, so I socialise with them. To be honest, it’s a narrow social circle’. The majority of participants (*n* = 8, 72.73%) emphasised the significance of room or dorm-based honours peer connections, with one student noting, ‘It’s convenient to make friends with my roommates because we live and have classes together’. Nonetheless, most participants (*n* = 6, 54.55%) indicated that in learning settings ‘it’s normal to study alone’ or ‘to stay connected with discussion group members often’.

Maintaining an elite identity came at the expense of various opportunities (*n* = 11, 100%), particularly limiting social interactions beyond the honours college (*n* = 9, 81.82%). One student mentioned ‘I feel I am always busy with academic stuff. Because of the workload, I don’t have time for student associations or clubs’. Another student experienced ‘loneliness and discomfort’ during their first year due to focusing solely on assessments, expressing ‘regret for not making new friends and socialising’. Additionally, to avoid elimination, students sacrificed opportunities to expand social networks. One student acknowledged ‘Sometimes I want to have fun or date. But once I think about elimination, it’s better to focus on academia instead of social or romantic relationship’. One participant stated that valuing studying over socialising was ‘all about making choices—you don’t study then you are out. I want to stay, so I work hard’.

Furthermore, competition and fear of elimination exerted pressure on students, leading all participants (*n* = 11, 100%) to feel threatened by re-streaming and to use better performers’ achievements as benchmarks for upward comparisons and motivation. The adoption of performance-avoidance goals was common (*n* = 10, 90.91%), leading to negative emotions such as anxiety, fear, and shame. One student described ‘I feel very anxious. Because it is possible that I were removed from the college, I am so fearful. It’s like I am always having my heart in my mouth’. Even netting high scores was not satisfying, with one saying, ‘I had a GPA of 4.3/5.0, but it’s a shame. I only ranked the third’.

Overall, these themes highlight the unique social dynamics and challenges faced by honours students in maintaining their elite status, including the trade-off between academic success and social engagement.

#### 3.2.2. Demoted Group

Feelings of otherness were evident for demoted students (*n* = 7, 100%). One interviewee expressed a sense of detachment from any group, ‘Both my current roommates and classmates in the new class are aware of my failure, making it difficult for me to connect with any of them’. One student highlighted weakened relationships with roommates, ‘As we no longer have classes together and they are constantly engrossed in studying, I feel a distinct contrast between them and myself in our academic lives’.

All participants (*n* = 7, 100%) recognised that seeking social support and expanding social networks were important. They actively engaged with peers outside of the honours programme and room/dorm, such as participating in student associations and clubs. One student described ‘Being part of the honours group became unbearable, as I struggled to fit in. However, joining a teaching support programme and a food club after re-streaming gave me a sense of belonging and happiness’. Another student found solace in talking about their experiences with peers in the student association, ‘Honours students cannot understand me. I have to talk my experience with my peers in the association so I feel better’.

Nonetheless, all interviewees (*n* = 7, 100%) exhibited minimal interest and reduced self-esteem following re-streaming, along with elevated levels of anxiety, depression, and shame. One student noted that ‘Even meeting the minimum requirement was impossible so I didn’t want to make efforts’. Another student expressed feelings of incompetence and hopelessness, ‘I always ranked the last in my class. I don’t want to study anymore’. Transferring to the non-honours track did not alleviate burdens but created concerns about the future (*n* = 5, 71.43%). One student expressed this sentiment, ‘With this reassignment, I am done. I am trapped with my demoted status’. Assessments were seen as external accountability (*n* = 5, 71.43%), which demotivated students, with one student saying, ‘The tests at university really stress me out. It feels like they’re always keeping an eye on us, and that makes me not want to do anything. I’ve been missing classes here and there because I just can’t stand going to class or dealing with assessment anymore’.

Performance-avoidance goals become demoted students’ focus in learning (*n* = 6, 85.71%). One described this mindset, ‘I don’t want to get lower scores than others. But the results are always disappointing. My scores became lower and lower’. Low scores reinforced their feelings of otherness, as one student put it, ‘There are so many *study bosses* in honours class, and I feel like a total underdog. I can’t compete with them. Honestly, I don’t fit in’.

## 4. Discussion

### 4.1. General Discussion

This study uses an ego-centric network approach and repeated measures before and after an academic sorting system to understand students’ study- and social-based ego networks. Employing mixed methods, this research uncovers insights into the network dynamics of students in different academic tracks. The honours group had more separated network circles and less varying social connections revealing distinct networks because of the selection/relegation process. Conversely, those who started and stayed in the non-honours class had more multiplex ties and more diverse social networks over time, indicating stronger ties. The demoted group had limited support and connections from their previous honours networks and exhibited negative responses towards re-streaming and assessment.

The findings support our hypotheses about longitudinal differences between students on divergent academic paths. Notably, the honours group had less dispersed social relationships (i.e., less variations in categories of friends in social settings) in Semester 2, with a preference for connecting with similar alters. This agrees with Hallinan and Sørensen’s study on a tracking system where students who were grouped based on academic abilities were more likely to engage with individuals within their track [19]. It also corresponds to more current and persistent findings of homophily in social networks where friend selection based on similarity (i.e., assortative pairing) [64] may occur in network formations [20,21,45]. According to our interviews, this procedure contributed to constructing an elite identity, enhancing students’ sense of superiority exclusive to the honours group. However, Domina and other scholars suggested that such socialisation might create a social closure [26,28] because students may draw boundaries against those outside the same ecology during social activities. According to research in the Chinese context, this phenomenon is particularly pronounced in elite environments in Chinese higher education, where competitive attitudes prevail and compromise positive peer relationships [18,33,36,38]. In the present honours track, competition emerged as the dominant peer norm, with honours students prioritising academic success over social connections.

Moreover, we found substantial difference in honours and non-honours groups’ network multiplexity during their first year. Honours students’ networks remained distinct in Semester 2, implying a compartmentalised student experience with separate close-knit connections in their elite setting. The literature suggests that this distinction could be attributed to lack of communication channels or a limited range of interactions between study and social situations [51,52]. For example, studies on the consequences of academic streaming indicate that when honours students associate predominantly with high-achieving peers within their study networks, there may be heightened pressure to excel academically and make comparisons among themselves [7,30]. As revealed by the interview data, honours learners would choose achievement over social engagement. Conversely, friendship circles of non-honours students overlapped more over time. This contrast, from the perspective of social capital in networks, may indicate that honours students could miss out on informal discussions or cross-border resource exchanges [52], while non-honours students would have a greater tendency to form deeper bonds and mobilise and exchange resources in diverse network situations [47,51,52].

Interestingly, both honours and non-honours groups had similar study network variations across categories of friends over time. As previous studies have suggested, this might be due to the cohort’s intention to take more opportunities for gaining and utilising academically useful information [65] and take advantage of the social capital in networks to achieve better outcomes [22,23,24,25]. Since this research was conducted in a selective university, it is plausible that these participants, who were high achievers through gaokao, leveraged social assets to search, obtain, and use resources for better performance [2,65]. These observations are also reflected by the interview data where students would establish strong ties with compatible performers in their study networks and be driven by such comparisons to achieve success. These findings align with motivational research on Chinese students whose achievement motivation involves the evaluation of academic performance relative to peers (i.e., performance goals) [34,39].

In terms of turnover rates, honours and non-honours students demonstrated comparable patterns in both semesters. According to ego network analysis [5,6,46], the presence of unreplaced networks suggests students maintained stable connections with a constant set of peers for knowledge sharing, influence, and support. In social environments, similar turnovers can be attributed to the stability of dorm and room settings that serve as important hubs for student interactions [16,41]. On the other hand, network replacement supports the notion of substitution in social network theory as students gradually adjusted to the higher education transition [45]. Particularly in study contexts, classroom environments may evolve as students chose different courses or engaged in group work, creating opportunities to form new ties. Social selection might be at play as students built new, learning-focused relationships with their available peers, replacing less stable bonds [6,66].

Most importantly, demoted students experienced a substantial disturbance in study-related networks because of elimination. Interviews revealed that the previous environment, characterised by elite status and pressure, hindered their development of supportive relationships because they felt like ‘outsiders’ [22] given their relatively low performance. Corresponding to previous findings [37,38,40], students experienced negative emotions (e.g., shame) from their relative underachievement due to the negative meaning of low performance in Chinese society. Further, the tag of relegation could contribute to a sense of not belonging to either the former honours group or current non-honours group. Thus, it is plausible that they had a greater tendency to reshuffle their network by connecting with similar peers who were not academically successful through social selection (i.e., assortative pairing) [64,67].

However, in Semester 2, the demoted students had more multiplex and wider social networks. In light of Wang and colleagues’ study [67], this might mirror their coping strategies for adversity and changing contexts by investing energy into contacts outside classrooms and seeking social support (e.g., emotional engagement). Through external resources, demoted students might have a better sense of engagement and involvement as a member of the university community. Whether these changing patterns would lead to improved learning outcomes and better psychological wellbeing merits future research.

### 4.2. Limitations

Several limitations should be considered when interpreting the results of this study. The participants are students with very high academic aptitude in a selective university, meaning the range of ability is quite restricted. Future research should replicate the present study with a broader range of students enrolled in different institution tiers or among younger students in secondary schools. This would provide a better understanding of how social network changes are influenced by high-stakes assessment streaming processes. It is important to acknowledge that although the findings on the demoted students may possess conceptual coherence, they must be approached with caution considering the limited sample size. Additional investigation utilising larger samples would be necessary in order to validate and generalise the results. Nonetheless, the preliminary findings from this exploratory study may serve as a foundation for future inquiries. Also, including students’ academic performance as an outcome variable in future research would enhance the understanding of relationships between achievement and students’ diverse networks. Further, although the present research managed to obtain network measures at the ego level, the analysis of ego networks was limited. It could be more insightful if future studies had data about network content (e.g., characteristics of the alters), strength (e.g., quality of social connections), function (e.g., types of exchanges or supports), and structure (e.g., ego’s position in a network) with different foci at the alter, ego–alter, and/or alter–alter levels to observe and explain network dynamics and their relationships to students’ wellbeing. Lastly, more nuanced results may surface if researchers could include measures of psychological wellbeing and conduct longitudinal investigations to examine the long-term impact of a streaming system on students’ social and learning experiences.

## 5. Conclusions

The present study is one of the first attempts to examine unintended social consequences of academic streaming on students. It contributes to the literature of social network analysis in education by utilising ego network measures and capturing relationships and their shifts among students along time. The results showed that students who started and stayed on the non-honours track had more multiplex ties and greater variation in social connections, indicating more stabilised relationships and stronger connections. Contrarily, honours participants inclined to socialise with similar peers and had less dispersed social ties, perhaps in response to the selection/relegation process. The impact on the social networks of demoted students is understandable and potentially threatening to psychological wellbeing.

These findings may possess clinical implications. Through acknowledging the impact of academic tracking on students’ social networks, educators and policymakers can make well-informed decisions pertaining to educational policies and interventions that seek to foster equitable opportunities for all students. Understanding the variables that influence favourable or unfavourable results can inform the creation of support mechanisms and resources tailored to the distinct needs of students in different academic tracks and with distinct movements.

## Figures and Tables

**Table 1:** Participant demographic information.

	Semester 1		Semester 2		Matched Rate	
Demographics	HO ^1^	NH ^2^	HO	NH	HO	NH
*Sex*						
Male	91	300	64 (4) ^3^	149	67%	52%
Female	50	301	31 (2)	138	33%	48%
*Faculty*						
Humanities	5	108	2	44	2%	15%
Social science	4	57	3 (1)	29	3%	10%
Engineering	11	148	10 (1)	63	11%	22%
Information technology	24	90	16 (1)	49	17%	17%
Agriculture, life science and environment	19	80	12	43	13%	15%
Medicine and pharmacy	25	71	13	39	14%	14%
Science	53	47	39 (3)	20	41%	7%
*Subgroup Total*	141	601	95 (6)	287	25%	75%
*Total*	742		382		51%	

^1^ HO = honours class; ^2^ NH = non-honours class; ^3^ The number of students who were restreamed from honours to non-honours classes is in the bracket.

**Table 2:** Descriptive statistics of network measures with effect sizes across time.

Network Measures	Honours (*n* = 89)		Non-Honours (*n* = 287)		Between-Group Difference Effect Size
	*M*	*SD*	*M*	*SD*	Cohen’s |*d*| ^1^
*Semester 1*					
Network size	4.16	2.06	4.41	2.08	0.12
Multiplexity	1.13	1.18	1.23	1.34	0.08
IQV ^2^ (study)	0.44	0.46	0.49	0.46	0.11
IQV (social)	0.35	0.45	0.43	0.45	0.18
*Semester 2*					
Network size	4.09	1.92	4.43	1.95	0.18
Multiplexity	1.13	1.16	1.49	1.23	*0.30* *
IQV (study)	0.54	0.46	0.55	0.45	0.02
IQV (social)	0.33	0.44	0.50	0.46	*0.37* **
*Between-Semesters Turnover*					
Study	0.55	0.27	0.53	0.26	0.08
Social	0.50	0.28	0.52	0.26	0.08
*Within-Group between Semesters Effect Size* (Cohen’s |*d*|)					
Network size	0.04		0.00		
Multiplexity	0.00		0.20 *		
IQV (study)	0.20 *		0.13 **		
IQV (social)	0.04		0.15 *		
Network turnover	0.19		0.04		

^1^ *Italics* indicates small to medium effect size (0.20 < *d* < 0.50); ^2^ IQV = Index of Qualitative Variation; * *p* < 0.05; ** *p* < 0.01.

**Table 3:** Social network measures of demoted students.

Case ID	Network Size		Multiplexity		Study IQV ^3^		Social IQV		Turnover	
	S1 ^1^	S2 ^2^	S1	S2	S1	S2	S1	S2	Study	Social
2362	8	9	2	1	0.96	0.84	0.96	0.96	0.67	0.40
2360	4	4	1	1	0.96	0.89	0	1	0.67	0.17
5212	4	4	1	1	0.89	0.89	1	1	0.57	0.29
4291	3	1	0	1	0	0	0	0	1.00	0.50
2976	3	5	0	3	0	0.75	0	0.75	0.86	0.71
3586	4	6	1	0	1	1	0	0	0.44	0.56
Mean	4.33	4.83	0.83	1.17	0.64	0.73	0.33	0.62	0.70	0.44
SD	1.86	2.64	0.75	0.98	0.49	0.62	0.51	0.49	0.20	0.20
Effect size ^4^	*0.33*		*0.22*		*0.29*		**0.64**		**1.14** *	

^1^ S1 = Semester 1; ^2^ S2 = Semester 2; ^3^ IQV = Index of Qualitative Variation; ^4^ *Italics* indicates small to medium effect size (0.20 < *d* < 0.50); **bold** indicates large effect size (0.50 < *d*); * *p* < 0.05.

## Data Availability

Publicly available datasets were analysed in this study. These data can be found here: https://doi.org/10.6084/m9.figshare.23528805.v1.
